# Cerebral Metastasis of Common Cancers

**DOI:** 10.3390/cancers13010065

**Published:** 2020-12-29

**Authors:** Johan M. Kros, Dana A. M. Mustafa

**Affiliations:** Laboratory for Tumor Immunology, Department of Pathology, Erasmus Medical Center, Dr. Molewaterplein 40, 3015 GD Rotterdam, The Netherlands; d.mustafa@erasmusmc.nl

Blood-brain barrier The incidence of brain metastasis has risen dramatically over the last decades and has equaled that of primary brain tumors [1]. At first sight this increase seems in contradiction with improved treatment options for cancer. Although screening strategies, early detection, and better surgical, pharmacological, and radiation interventions have led to better survival rates for many cancer types [2], incidences of cerebral metastasis did not drop concurrently. Apart from the extension of life expectancy, leading to increased numbers of cancer cases, there are other explanations for this contradiction. The successful treatment regimens for breast cancer have led to better control of the primary tumors but the time window for potential development of brain metastases increased. Further, there may be effects of chemotherapy-induced selection of particular tumor clones with a higher affinity for the brain. There are several trials conducted on the effects of particular drugs on established brain metastasis [3,4]. The prevention of tumor cells reaching the brain is another current topic [5,6]. In most studies tumors are characterized, traditionally, by the organ of origin, by classic histology, and immune-phenotyping. In some recent trials, however, the characterization is substituted by genetic parameters [7,8]. An important issue is the similarity of the brain metastasis with its parental tumor. In fact, an even more essential question is whether particular metastases arise from the primary tumor, or represent seeding from another metastasis. It seems that driver mutations usually remain to be present in the metastatic tumor cells, but that the latter may differ significantly in additional genetic make-up. Then follows the important question about suitable chemotherapy for the metastatic tumor cells.

It has been known for a long time that cancers arising in various organs have different affinities for the brain [9]. Lung cancers usually give rise to brain tumors relatively soon after the diagnosis is made. Brain metastases originating from breast cancers usually arise late in the course of the disease. Triple negative breast tumors usually disseminate quickly to various organs, including the brain. Other tumors with well-known predilection for cerebrum are melanomas and, to a lesser extent, renal cell carcinomas. In order to study the essentials of each individual step of the metastatic cascade, various experimental models have been developed [10]. Various cell lines, to be used as representatives of different cancers are available, by now [11]. Apart from the molecular level, also biomechanical forces are operative at the sites where tumor cells land [12]. Repertoires of gene expression and molecular pathways specifically involved in passage through the blood-brain barrier (BBB) are topics of current research [13,14]. The effects of immune cells on the primary tumor may also be relevant for the rise of brain metastases: T-cell interactions with tumor cells change the expressional repertoires of tumor cells that seem to facilitate their capacity to pass the BBB [15]. While this effect was found in estrogen receptor-negative breast cancers, no such mechanism seems to be prominent in lung cancer. The expression of sets of microRNAs are operative in the metastatic cascade to the brain as well [16] and micro-environmental factors and interactions between the residential and metastatic cells are topics of current research [17,18]. Part of the molecular mechanisms underlying the passage of tumor cells through the BBB implicate motility of the tumor cells [19]. Another part involves extracellular vesicles, steered by microRNAs, that breach the intactness of the BBB [20,21,22]. Once the cancer cells have passed through the BBB they enter the perivascular space of the cerebral blood vessels, where they may reside for variable lengths of time to ultimately migrate into the brain tissue [23]. The creation of, and interaction with, newly formed blood vessels plays a large role in the progress of invasion. The outgrowth of tumor cells in the brain is dependent on the expressional repertoire of the tumor cells and their interactions with cells in the brain micro-environment, including representatives of the local immune response [24,25]. The parts played by either the innate or the acquired immune response on outgrowth are topics of current explorations [26]. The innate immune system represented by perivascular microglia and macrophages interact with the tumor cells, and immune modulation is being scrutinized as prophylaxis for the rise of brain metastases [27]. For the development of therapeutic or preventive strategies it is important to delineate and scrutinize the various stages of brain metastasis, and their diversity among and between various cancer types.
